# The Development of Feeding and Eating Disorders after Bariatric Surgery: A Systematic Review and Meta-Analysis

**DOI:** 10.3390/nu13072396

**Published:** 2021-07-13

**Authors:** João Victor Taba, Milena Oliveira Suzuki, Fernanda Sayuri do Nascimento, Leandro Ryuchi Iuamoto, Wu Tu Hsing, Leonardo Zumerkorn Pipek, Luiz Augusto Carneiro-D’Albuquerque, Alberto Meyer, Wellington Andraus

**Affiliations:** 1 Faculty of Medicine FMUSP, University of São Paulo, São Paulo 05403-000, Brazil; joao.taba@fm.usp.br (J.V.T.); milena.suzuki@fm.usp.br (M.O.S.); fernanda.nascimento@fm.usp.br (F.S.d.N.); leonardo.pipek@fm.usp.br (L.Z.P.); 2 Center of Acupuncture, Department of Orthopaedics and Traumatology, University of São Paulo, São Paulo 05403-000, Brazil; leandro.iuamoto@gmail.com (L.R.I.); consultoriowitta@gmail.com (W.T.H.); 3 Department of Gastroenterology, Hospital das Clínicas, HCFMUSP, São Paulo 05403-000, Brazil; profluizcarneiro@gmail.com (L.A.C.-D.); wellington@usp.br (W.A.)

**Keywords:** bariatric surgery, feeding and eating disorders, binge-eating disorder, food addiction, night eating syndrome

## Abstract

Background: Patients in the postoperative period following bariatric surgery are at risk of developing eating disorders. This study aims to analyze the relation between bariatric surgery and the development and recurrence of eating disorders. Material and methods: A literature review was carried out on 15 November 2020. Fourteen studies that met the eligibility criteria were included for qualitative synthesis, and 7 studies for meta-analysis. Results: The prevalence of eating disorders in the postoperative period was 7.83%, based on the 7 studies in the meta-analysis. Binge eating disorder alone was 3.81%, which was the most significant factor, and addressed in 6 of these studies. Conclusion: The investigated studies have significant methodological limitations in assessing the relation between bariatric surgery and eating disorders, since they mostly present data on prevalence. PROSPERO CRD42019135614.

## 1. Introduction

Bariatric surgery has been one of the main and most effective treatments for obesity. In 2011, approximately 340,000 cases of bariatric surgery were registered worldwide, with the United States (USA) and Brazil being the two largest performers of this procedure (101,000 and 65,000, respectively, accounting for 48.8% of a total of 50 countries studied) [1]. In 2018, 252,000 surgeries of this type were performed in the USA alone. Sleeve gastrectomy (SG) became the most performed procedure in the USA in 2018 (61.4%), with an increase of over 451% since 2011 (17.8%). Roux-en-Y Gastric Bypass (RYGB) was the second most performed procedure in the USA in 2018 (17.0%), despite decreasing by 25.9% since 2011 (36.7%) [2]. 

This shows a rapid increase over the last decade, highlighting the need to better understand the outcomes in the post-operative period, such as non-preexisting eating disorders. Data regarding its incidence in the postoperative period are scarce due to the lack of follow-up, screening, or standardization in the evaluation of these comorbidities [3].

The current literature provides no significant number of publications that address the role of bariatric surgery as a factor that can lead to the development of new cases of eating disorders, especially in patients with no previous history of them. Current studies show the need for better understanding, because they do not consider surgery in depth as a potential risk factor: Opozda M et al., Williams-Kerver GA et al. and Brode CS et al. verify the significance of recurrence and new cases of eating disorders [4,5,6]. Meany G et al. notes the possibility of the emergence of new pathological eating behavior in the postoperative period for patients with symptoms of compulsive eating [7].

The added value of this study is the fact that, to the extent of our knowledge, this is the first systematic review with a meta-analysis that considers several eating disorders, with data screened from 1985 to 2020. Our aim is to analyze the relation between bariatric surgery and the development or recurrence of eating disorders in patients with or without pre-existing history. Thus, this review highlights with qualitative and quantitative data an underrated topic in the current literature, identifying its limitations in such a way as to guide and suggest ideas for new research.

## 2. Material and Methods

This systematic review was carried out in accordance with the items of Preferred Reports for Systematic Reviews and Protocol Meta-Analysis (PRISMA-P) [8]. This study was registered by the Prospective Register of Systematic Reviews (PROSPERO, identification code CRD42019135614) before the research was carried out.

Drafting of the research question was based on the PICO strategy [9], considering patients in the postoperative period of bariatric surgery (Patient or Problem); psychiatric assessment methods for the development of eating disorders (Assessment); there is no standard comparator to be considered in this study (Control or Comparison); all outcomes available in the literature were considered in the analysis (outcome).

### 2.1. Eligibility Criteria

#### 2.1.1. Types of Studies

The articles were selected from their titles and abstracts according to their data relevance and regardless of their publication status.

The following study designs were considered: randomized and non-randomized controlled clinical trials, prospective and retrospective cohorts, cross-sectionals, and case controls. Reports and case series, reviews, letters to editors, research protocols, and conference proceedings were not considered.

#### 2.1.2. Types of Participants

Study participants were adult patients in the postoperative period of bariatric surgery, evaluated or treated in any type of institution.

#### 2.1.3. Types of Variables/Parameters Analyzed

Data related to the authors, date and location (country) of the publication, type of study, types of bariatric surgery, and psychiatric evaluations performed were collected and arranged in tables. Data were also collected regarding the number of patients analyzed in the study, sex, age, pre and postoperative body mass index (BMI), type of disorder and pre and postoperative symptoms, reported limitations, objectives, and conclusions of all studies.

### 2.2. Exclusion Criteria

Studies were excluded if: (1) they did not present data related to the number of patients diagnosed with eating disorders or who are restricted to symptoms or scores; (2) were incomplete unpublished articles or with full text inaccessible to the authors; (3) were in languages other than English and Portuguese.

### 2.3. Literature Revision

The survey was conducted on 15 November 2020, without language or date restrictions, in the following databases: Medline (via PubMed)—www.pubmed.com, accessed date: 15 November 2020; EMBASE—www.embase.com, accessed date: 15 November 2020; Cochrane Library—www.thecochranelibrary.com, accessed date: 15 November 2020; Database of the National Institute of Health. In addition, a manual search of theses, meetings, references, study records, and contact with specialists in the field was carried out.

#### 2.3.1. Search Strategy

The keywords were used equally in all databases, respecting their heterogeneities (for example, terms “Emtree” and terms “MeSH” were mapped in Embase and Medline, respectively).

The keywords were: “bariatric surgery”, “feeding and eating disorders”, “anorexia nervosa”, “bulimia nervosa”, “binge-eating disorder”, “pica”, “food addiction”, “night eating syndrome”.

The search strategy was: (Bariatric Surgery) AND ((Feeding and Eating Disorders) OR (Anorexia Nervosa) OR (Bulimia Nervosa) OR (Binge-Eating Disorder) OR (Pica) OR (Food Addiction) OR (Night Eating Syndrome)).

#### 2.3.2. Data Extraction

The data for each study were extracted independently by three authors (JVT, MOS, and FSN). Disagreements were resolved by consensus. If no consensus was reached, a fourth author (AM) was consulted.

All studies were analyzed according to their titles and abstracts, according to inclusion and exclusion criteria. If the eligibility criteria were met, the full text was extracted. All studies with full text evaluated are described in the “Results” section.

Missing data were clarified by contacting the authors directly.

#### 2.3.3. Data Validation

Three authors (JVT, MOS, and FSN) carried out the data validation through the discussion of the selected studies. If no consensus was reached, a fourth author (AM) was consulted.

The bias risks for the studies were assessed using the criteria of the Study Quality Assessment Tools | National Heart, Lung, and Blood Institute (NHLBI) [10]. Intervention-type studies were analyzed using the Cochrane Back Review Group (CBRG) guidelines [11].

All selected studies were considered.

### 2.4. Statistical Analysis

The characteristics of study participants were presented as means, minimum and maximum values for quantitative variables, and as frequencies and percentages for qualitative variables. The prevalence values and 95% confidence intervals were calculated using the Wilson method due to small frequency values [12].

The meta-analysis was developed to evaluate the results of the systematic review. To assess the global heterogeneity between the studies, Cochran’s Q test was calculated, as well as the I2 (percentage of variation). A forest-plot was used to present the results of the studies’ association measures and their respective 95% confidence intervals.

Statistical analyses were performed using the Stata/MP 14.0 software for Windows.

For the statistical analysis, we evaluated the total number of the following data for both before and after the interventions: patients, patients with any sort of eating disorder and patients with each type of eating disorder separately. Also, we considered the type of study and the type of surgery.

## 3. Results

### 3.1. Search Flow

**Figure 1:**
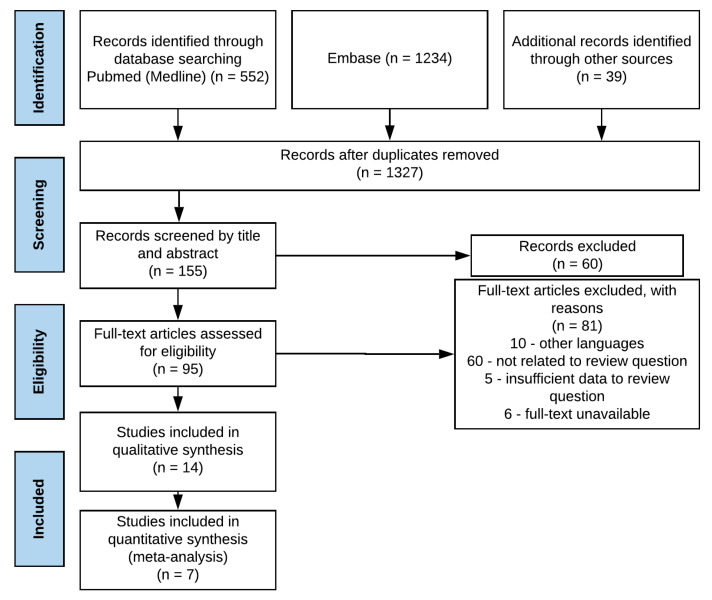
Search flow.

The electronic search found 1825 results for the keywords used. After removing 498 duplicates, 155 potentially eligible studies were identified. Of these, 60 studies did not fulfil the inclusion criteria and 81 did not fulfil the exclusion criteria. Only 14 studies were included in qualitative synthesis and 7 in meta-analysis (Figure 1).

### 3.2. Quality of Evidence

**Figure 2:**
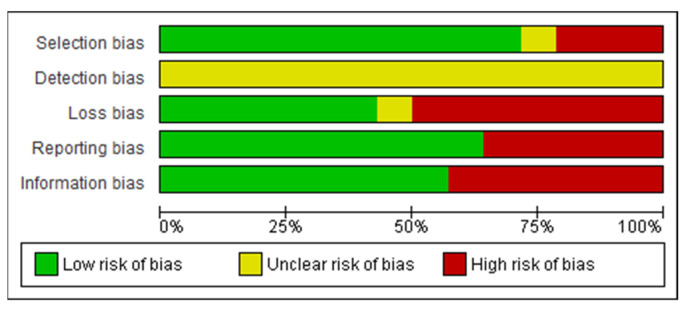
Graph of risk analysis of general bias in articles.

**Figure 3:**
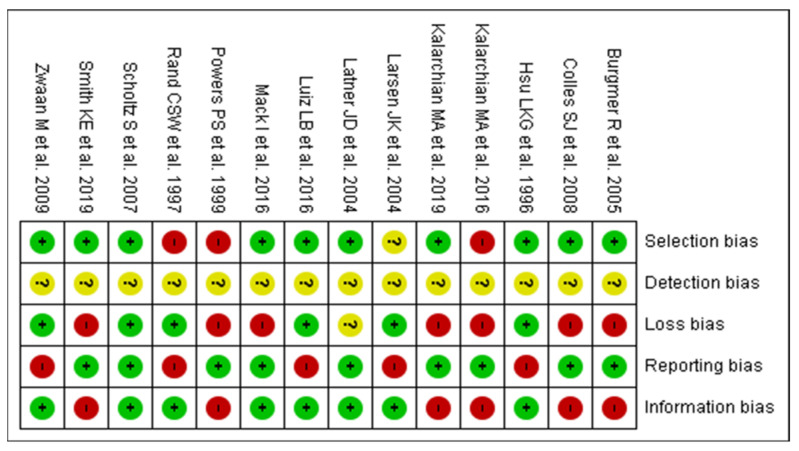
Summary of risk analysis of general articles bias [13,14,15,16,17,18,19,20,21,22,23,24,25,26].

After reading the articles included in the systematic review, the following factors were analyzed to determine the level of evidence: study design and selection, detection, loss, and reporting and information bias. The summary of the risk of bias analysis for each of the included articles is shown in Figure 2 and Figure 3.

All the articles analyzed presented uncertain detection bias, since they did not explicitly inform the blinding method of the study in the methodology.

Mack I et al. [13] was the only clinical trial analyzed. The study showed low bias in selection (allocation concealment), reporting (selective reporting), and attrition (incomplete outcome data). The biases of performance (blinding) and detection (blinding regarding outcome assessment) were uncertain. As for selection bias (random sequence generation), it had a low risk of bias.

In the case-control of Rand CSW et al. [14], it was not possible to prove the consistent application of eligibility criteria for the selection of the sample. However, despite the high selection and reporting bias, it had a low loss and information bias, which allowed for a comparison of the pre and postoperative periods of the same individual.

All 4 cross-sectional studies showed high reporting bias due to the use of self-reported questionnaires without following the sample to re-evaluate results. However, all showed low information bias. In addition, the loss bias was classified as low due to the lack of follow-up in cross-sectional studies. Larsen JK et al. [15] showed uncertain selection bias, as they did not previously specify the inclusion and exclusion criteria for patients, making it unclear how this selection was made.

All 8 cohorts had low reporting bias and 6 of them had low selection bias. In addition, one study stood out for its low loss and information bias: Scholtz S et al. [16] maintained at least 80% of the sample during the follow-up period. Latner JD et al. [17] presented uncertain loss bias, since the reviewers did not obtain enough data to determine the percentage of loss from follow-up.

### 3.3. Study Characteristics

All included studies were complete and had been published. Doubts about the available data were supplemented by contacting the respective authors. The demographic characteristics collected are shown in Table 1; the main changes, conclusions and results are made available in Table 2, Table 3 and Table 4. The reported limitations are available in Table 5. 

All studies elected a total of 5774 participants. Considering that Colles SJ et al. [18] partially informed the gender distribution of the sample, 4066 (71%) women and 1664 (29%) men were evaluated. The average age of the participants analyzed was 42.8 years (range 38.30–49.20); and the mean BMI before and after surgery was, respectively, 48.30 kg/m^2^ (range 44.30–54.10) and 35.6 kg/m^2^ (range 26.80–45.40).

Of the 14 selected articles, 5 (35.71%) were recently published—later than 2015, 4 (28.57%) studied more than one type of bariatric surgery, and 7 (50%) applied more than one method of assessing eating disorders.

Only three articles documented detailed data on the postoperative development of new types of eating disorders for previously healthy patients or those with another eating disorder. Hsu LKG et al. presented the general psychiatric history of the sample and classified it according to the types of eating disorders-or their absence-before and after surgery: 2 patients with a history of NES converted their disorder to BED (Binge-Eating Disorder) or BN (Bulimia Nervosa) after surgery [19]. Luiz LB et al. documented the prevalence of BED in the preoperative period and 1 year after surgery. In the postoperative period, 10 individuals (7.58%) met the criteria for BED by BES and, of these, only 3 were new cases [20]. Kalarchian MA et al. (2016) had a 3-year follow-up. After 2 years of surgery, the incidence was zero, however, after 3 years, 1 patient (0.9%) without a previous history of BED developed the disorder [21].

Two articles presented only symptomatologic data or referring to questionnaire scores instead of data referring to the incidence of eating disorders diagnosed at some point in the study, either in the pre or postoperative period. In Powers PS et al., the frequency of vomiting episodes was reported by patients in the postoperative period—46 occasional (79%) and 19 weekly (33%) [22]. De Zwaan M et al. reported only the presence of eating symptoms in the postoperative period, including those related to other disorders—15 Loss of control eating (LOCE), 44 vomiting episodes and 7 NES symptom—without precisely diagnosing any disorder [23]. 

Only two studies used a differentiated methodology from the others to analyze the impact of postoperative eating disorders. Larsen JK et al. used 3 different groups, preoperative period/pre-surgery, short-, and long-term after surgery, establishing the incidence of BED in each (55.9%, 31.9%, and 37.4%, respectively) [15]. However, when using three different groups, it was not possible to establish whether new disorders were developed, despite demonstrating that there is less incidence after the operation. Luiz LB et al. analyzed variations in the intensity of symptoms in addition to the prevalence of BED. After surgery, 18 patients (13.63%) had an increase in intensity and 105 patients (79.54%) had a decrease [20].

**Figure 4:**
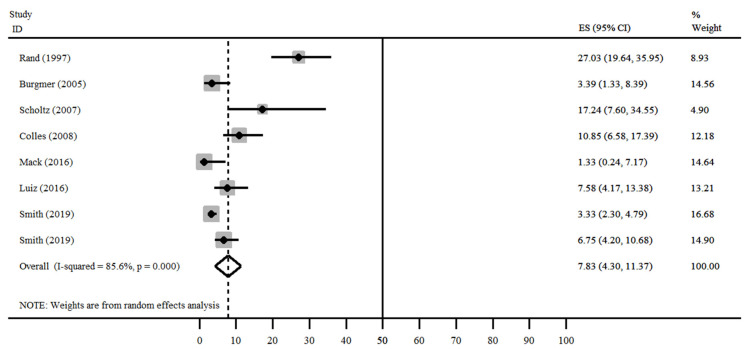
Total prevalence of disorders in the postoperative period [13,14,15,16,17,18,19,20,21,22,23,24,25,26].

**Figure 5:**
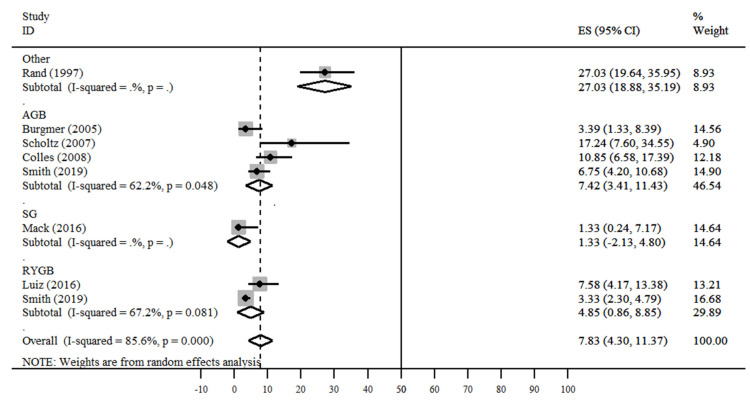
Prevalence of disorders in the postoperative period by type of surgery [13,14,15,16,17,18,19,20,21,22,23,24,25,26].

**Figure 6:**
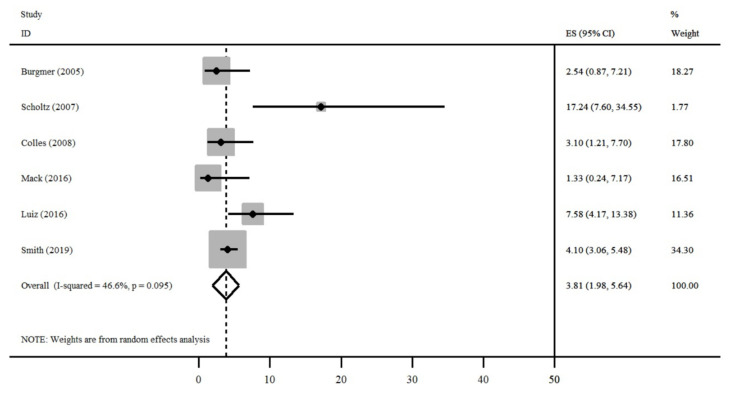
Prevalence of BED (Binge-Eating Disorder) in the postoperative period [13,14,15,16,17,18,19,20,21,22,23,24,25,26].

The results of the meta-analysis (Figure 4, Figure 5 and Figure 6) demonstrate that the studies showed a high degree of heterogeneity (I2 = 85.6%, *p* < 0.001). For all studies, the overall prevalence of eating disorders was 7.83% (95% CI = 4.30–11.37%). Three studies showed values lower than 5% (Mack I et al., Burgmer R et al. and Smith KE et al.), Smith KE et al. presented a lower confidence interval than the others (95% CI = 2.30–4.79%) [13,24,25].

In the stratified analysis by surgery type, only Mack I et al. [13] presented the results of SG surgery. RYGB groups showed homogeneity (*p* = 0.081) and prevalence values between 3.33–7.58%. In addition, the studies of the AGB group (*p* = 0.048) were homogeneous, with prevalence values between 3.39% and 17.24%. Finally, Rand CSW et al. did not specify the type of surgery performed, being classified as “other”, with a prevalence of 27.03% [14].

**Figure 7:**
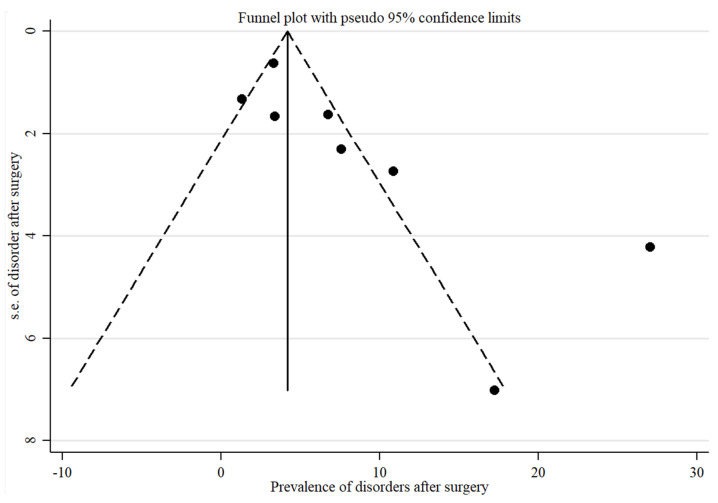
Analysis of publication bias.

A funnel-plot was constructed considering all studies of the meta-analysis to assess publication bias (Figure 7). Three studies were found outside of the expected standard error: Mack I et al., Rand CSW et al. and Colles SJ et al., as also observed by the confidence intervals shown in Figure 6 [13,14,18].

#### Types of Evaluation

The types of evaluations found in this study were the following psychiatric questionnaires to assess the development of eating disorders and their variations applied to the mentioned participants: (1) Eating Disorders Examination (EDE), (2) Three Factor Eating Questionnaire (TFEQ), (3) Medical Outcomes Study Short Form-36 Health Survey (SF-36), (4) Binge-Eating Scale (BES), (5) Eating Disorder Inventory (EDI), (6) other methods.

The “other methods” group includes tools that were referred less than 3 times throughout the studies: Structured Interview for Eating Disorders, Patient Health Questionnaire, Dutch Eating Behavior Questionnaire, Dutch Fat Consumption Questionnaire, Satiety-Questionnaire, Obesity Psychosocial State Questionnaire, Questionnaire on Eating and Weight Patterns, Cancer Council Victoria Food Frequency Questionnaire, Beck Depression Inventory, Multidimensional Body Self-Relations Questionnaire, Self-evaluation of LOCE and BED, Alcohol Use Disorders Identification Test, Binge Scale Questionnaire, Eating Attitudes Test, Bulimia Cognitive Distortion Scale, Body Parts Satisfaction Questionnaire, Structured Clinical Interview, Structured Interview for Anorexia and Bulimia nervosa.

EDE is a tool to help diagnose eating disorders in general. It addresses various food and self-image issues (e.g., objective binge eating, subjective binge eating, and LOCE) through self-reported questionnaires and criteria [27]. Berg K.C. et al. found its reliability for specific populations or diseases (e.g., women with BN), but points to the need for further studies on its psychometric properties and efficacy in more generalized samples [28].

The TFEQ is a scale that measures three types of eating behavior: cognitive restraint, uncontrolled eating and emotional eating. A psychometric analysis carried out in 2009 found reliability in the questionnaire [29] and, a decade later, Bryant E.J. et al. reported the popularity of the questionnaire and reinforced its ability to identify pathological eating behaviors related to restriction and disinhibition [30].

SF-36 addresses issues of quality of life and physical and mental health factors. Higher scores indicate healthier results. Although these factors are not specific for eating disorders, the tool has a significant consistency, reliability, and validation [31,32].

BES is a specific scale to measure binge eating behavior that can be used before and after bariatric surgery. Its isolated use is not sufficient for the diagnosis of BED, being only a clinical aid. In addition, a significant number of false-positive results should be considered for screening the disease in candidates for surgery [33].

EDI is an extensive tool composed, in its most recent version (EDI-3), by 91 questions that quantify eating behaviors and assist in the diagnosis of eating disorders. Its most recent version was evaluated with excellent sensitivity and specificity, good discriminatory validation, and satisfactory consistency [34].

All variations and translations of these tools were considered during our analysis, although they did not receive their own ratings.

It is important to note that there is a lack of standardization of several elements that makes it difficult to collect highly reliable data: (1) there is more than one guideline for the diagnosis of eating disorders, DSM (Diagnostic and Statistical Manual of Mental Disorders) [35] and ICD (International Statistical Classification of Diseases and Related Health Problems) [36]; (2) the use of the type of questionnaire applied is at the discretion of the clinician; (3) the questionnaires show only dietary symptoms and other variables, serving only as a diagnostic aid; (4) there is no clear guideline for the use of these tools in the pre- or postoperative period [37,38]; (5) among all the screened articles, there was only one adapted instrument for bariatric patients (EDE Bariatric Surgery Version—EDE-BSV [39]). Moreover, (6) some studies used only self-report questionnaires for assessment, while others used clinical interviews, or both methods, meaning that there was no assessment standardization throughout the studies. Despite that, all of them were considered by the authors.

## 4. Discussion

Bariatric surgery incidence is increasing considerably, making its consequences significant, such as the incidence and recurrence of eating disorders. This systematic review analyzed 14 articles, 7 of which were eligible for meta-analysis, which included data on bariatric surgery and eating disorders in the postoperative period, with different proposals for evaluation methods.

The methods analyzed are heterogeneous. The most part of instruments approach the individual in a more generalized way, but there are also significant differences between some of them: the TFEQ identifies three types of pathological eating behaviors only related to restriction and disinhibition [29,30]; and the SF-36 can be used to assess quality of life and mental and physical health factors [31,32].

Parker K. et al. compared the effectiveness of EDE, TFEQ, SF-36 and other evaluation methods in the context of bariatric surgery, both in candidates and in patients [40]. The results of the studies suggest that EDE is the most appropriate to be used in the pre- and postoperative context of surgery. In addition, the tools in their adapted forms showed more reliable results [40,41].

Despite this, the studies analyzed used several questionnaires, 5 of which were more significant and prevalent. This heterogeneity implies the absence of a gold-standard method for assessing eating disorders, leaving the examiner to choose the questionnaires. Consequently, during the screening process, many of the excluded studies presented only symptom or score data, without presenting definitive diagnoses, due to the multiplicity of guidelines (ICD and DSM). Thus, it is difficult to identify the disorders and, as a result, to characterize the onset or recurrence of the disorder after surgery.

From the studies that presented with definitive diagnoses it was possible to make the qualitative analysis, considering the time of follow-up of the studies and relating them with the results presented. We observed that, regardless of the type of surgery performed, the operation considerably reduces the rates of eating disorders and symptoms. However, in relation to the prolonged postoperative period, some studies have presented conflicting data.

Smith KE et al. presented, after 7 years, an increase in the prevalence of disorders and symptoms: from 2.1% to 4% of BED and from 24.6% to 26.4% of LOCE [25]. A hypothesis for the increase of the indices may be in the high rate of losses and selection bias, due to the extensive follow-up. On the other hand, Kalarchian MA et al. (2019) reported, in 7 years, a decrease in the prevalence of eating disorders to 0%, even though it presents a loss rate similar to the other two studies [26]. This can be explained by the exclusive use of a clinical interview based on DSM-IV for diagnostic closure; and use of the interview concomitant with the application of SF-36, a tool focused on quality-of-life issues and little specialized in eating disorders, as a method to measure the evolution of the participants. These factors may have contributed to a limitation of the diagnosis, underestimating the final prevalence, and overestimating the clinical improvement.

In addition, among the 12 studies that reported any symptomatic data, only Scholtz S et al. did not show symptoms rates higher than the rates of disorders. This may have occurred due to the small sample size (24 patients), which may generate bias due to the select sample of patients who already had symptoms [16].

The articles used for the analysis have a greater amount of data related to symptoms compared to the disorders themselves. This can be attributed to the large number of non-standard questionnaires that were applied. De Zwaan M et al. used two types of questionnaires to assess the presence of BED in its sample, obtaining two different results: 14 patients diagnosed with BED by QEWP (Questionnaire on Eating and Weight Patterns) and 15 by EDE-BSV [23]. Thus, we can infer that, depending on the questionnaire used, there may be a higher rate of underdiagnosis and less patient care.

Therefore, depending on non-standard methods, it was difficult to determine the relation between bariatric surgery and the development of eating disorders. This possibility, however, should not be ruled out, since other types of surgery have already been shown to be associated with the development of mental disorders, such as cardiac surgery, gastrointestinal cancer surgery, or liver transplantation [42,43,44].

In addition, bariatric surgery, specifically, also has its participation, acting for the development of impulsive disorders, such as alcohol and substance abuse [45]. The pathophysiology of this type of behavior is the same for eating disorders, with increased activity in the reward system, due to greater awareness of dopaminergic activity in the region of the nucleus accumbens [46,47]. So, just like substance abuse, there must be a greater risk for the development of disorders such as BED, NES and associated symptoms (LOCE, OBE, SBE).

Unlike the relationship shown between bariatric surgery and substance abuse disorders, establishing the same for eating disorders is a difficulty. This was the purpose of this systematic review, for the high prevalence of these disorders in bariatric surgery patients [48]. The Symptom-Checklist-K-9 is a promising attempt to develop a validated questionnaire [49,50], but there is still a lack of standardization of questionnaires and evaluation methods throughout the literature, reflecting the lack of a gold-standard method, thus it was not possible to make this analysis. Therefore, a standardized methodology is necessary for more studies to be carried out, as also stated by de Zwaan M et al. [23], making it possible to analyze the cause-and-effect relationship between bariatric surgery and disorders.

### Study Limitations and Methodologies

The most common limitations reported in the studies analyzed involve the use of a cross-sectional study design; the total duration of the study; the sample size and the use of self-reported questionnaires (Table 5).

Despite the limitations, some studies have presented different methodologies. Hsu LKG et al., Luiz LB et al. and Kalarchian MA (2016) et al. provided data on the incidence of eating disorders in the postoperative period, which is necessary for an adequate assessment of the relationship between bariatric surgery and the development of these disorders [19,20,21]. For analyzing more than one type of disorder, Hsu LKG et al. made the incidence data available, making it possible to observe in detail the number of conversions between the disorders [19].

In order to better assess the relation between bariatric surgery and the development of eating disorders, we propose that future studies expose data relating to the pre and postoperative periods, reporting the number of new cases, remissions and conversions. We also suggest the use of randomized, single-arm trials with patients undergoing bariatric surgery. The analysis would be accompanied by a historical control group, with participants eligible for surgery who chose not to perform it. Thus, it would be possible to establish an association or risk factor between surgery and the development of eating disorders.

Among the limitations of our systematic review is that we did not consider the possible interference of comorbidities both before and after surgery. In addition, there may be differences in results due to the use of different versions of diagnostic manuals between studies, since the articles analyzed date from 1996 to 2019. There was a lack of standardized instruments validated for bariatric patients, possibly limiting the generalization of our results to the bariatric population.

For a greater quality of evaluation, we emphasize the need for further studies to find alternatives to self-reported questionnaires and standardized instruments, in addition to more objective diagnostic methods. Functional magnetic resonance imaging, for example, has proved to identify neural networks involved in eating disorders, although its study is still incipient. More research is needed in this area, but they can be a way to standardize more objective diagnostic methods [51].

## 5. Conclusions

The current literature has a greater focus on bariatric surgery as a treatment for obesity, but it has important methodological limitations to evaluate its relationship with the development of eating disorders. An example of this are the studies analyzed, which mostly present only prevalence data. In this review, the total prevalence of eating disorders was 7.83%, based on the 7 studies in the meta-analysis. Considering only BED, which constitutes 6 of these studies, it was 3.81%, being the most significant disorder. This relevance can be attributed to the greater number of studies that approach it, constituting 13 studies out of a total of 14 from our review.

Even with postoperative prevalence below 10%, such disorders can significantly influence prognosis and weight loss. However, the role of surgery in the development of eating disorders or in the evolution of pre-existing ones is not well established. Thus, a rigorous and standardized psychiatric assessment is necessary, actively seeking to identify these disorders, which may be against surgical indication. Furthermore, to establish an association and risk assessment, more research is needed in this area, using more appropriate models, as suggested in this review.

## Figures and Tables

**Table 1:** Demographic characteristics of studies.

Author, Publication Date and Country	Number of Patients	Mean Age-Years (SD)	Sex (%)	Mean BMI before and after Bariatric Surgery-kg/m^2^ (SD)	
Larsen JK (2004), Netherlands [15]	Total: 250 Pre-group: 93 Post-group: 157 (short-term: 48, long-term: 109)	39.6 [range 22–61]	29 M (72.5) 221 F (27.5)	46.5 [range 37–67]	45.4 [range 36–63]
de Zwaan M (2009), Germany [23].	59	44.5 (9.9)	9 M (15) 50 F (85)	51.3 (9)	32.6
Mack I (2016), Germany [13].	75	49.2 (11.6)	27 M (36) 48 F (64)	48.7 (8.4)	37.1 (8.1)
Hsu LKG (1996), USA [19]	24	39.7 (8.6)	0 M (0) 24 F (100)	48.8 (8.1)	34.1 (7.7)
Latner JD (2004), New Zealand [17]	65	39.5 [range 19–67]	0 M (0) 65 F (100)	54.1 (10.2)	34.1 (8.5)
Colles SJ (2008), Australia [18]	173 (initial) 129 (final)	45.2 (11.5)	26 M (20) (final) 103 F (80) (final)	44.3 (6.8)	35 (6)
Scholtz S (2007), UK [16]	29	39 (9)	1 M (3.5) 28 F (96.5)	45 (7)	-
Smith KE (2019), USA [25]	Total: 2156 RYGB: 1640 (initial); 812 (final) AGB: 516 (initial); 237 (final)	45.66 (11.32)	517 M (24) 1639 F (76)	47.06 (7.36)	Total: 35.17 (6.44) RYGB: 33.82 (6.56) AGB: 40.23 (6.47)
Powers PS (1999), USA [22]	116	39.6 (9.3)	20 M (17) 96 F (83)	53.4 (10.9)	40.7 (9.5)
Kalarchian MA (2019), USA [26]	Total: 173 (initial); 98 (final) RYGB: 104 AGB: 69	RYGB: 45 (median) [IQR 34-53] 21–68 (range) AGB: 47 (median) [IQR 40–54] [range 23–67]	31 M (18) 142 F (82) RYGB: 20 M (19.2) 84 F (80.8) AGB: 11 M (15.9) 58 F (84.1)	RYGB: 46.9 (median) [IQR 43.1–52] [range 36.1–76] AGB: 43.5 (median) [IQR 40.8–46.7] [range 33.5–65.8]	-
Burgmer R (2005), Germany [24]	149	38.8 (10.3)	47 M (32) 102 F (68)	50.9 (8.1)	38.6 (6.8)
Rand CSW (1997), USA [14]	Total: 2208 Control group: 2097 Experimental group: 111	Control group: 52.8 (19.8) Experimental group: 44.6 (10.4)	Control group: 887 M (42.3) 1210 F (57.7) Experimental group: 8 M (6.9) 103 F (93.1)	-	Control group: 24.9 (4.9) Experimental group: 28.7 (6.4)
Luiz LB (2016), Brazil [20]	132	38.27 (10.07)	27 M (20.5) 105 F (79.5)	48.31 (7.92)	31.74 (5.7)
Kalarchian MA (2016), USA [21]	165	46 (median)	35 M (18.9) 130 F (81.1)	44.8 (median)	-

M: male; F: female; FA: food addiction. BED: binge-eating disorder. BE = binge eaters. LOCE = loss of control eating. RYGB = Roux-en-y gastric bypass. AGB = adjustable gastric banding. SG = sleeve gastrectomy.

**Table 2:** Studies objectives and conclusions.

Author, Publication Date and Country	Study Objectives	Study Conclusion
Larsen JK (2004), Netherlands [15].	“To examine short and long-term eating behavior after laparoscopic adjustable gastric banding (LAGB) and the relationship of binge eating with weight and quality of life outcome.”	There is an improvement in short- and long-term post-bariatric disorders. The diagnosis and treatment of BED in the post is essential for a better prognosis.
de Zwaan M (2009), Germany [23].	“(1) To provide a detailed description of the postoperative eating behavior of patients who had undergone RYGB and to determine which eating behaviors might be labeled non-normative or problematic; (2) to determine whether preoperative eating disorders might be associated with non-normative postoperative eating behaviors; (3) to determine the association of postoperative non-normative eating behaviors with postoperative eating-related and general psychopathology; and (4) to assess the association of preoperative and postoperative eating behaviors with the weight outcome.”	Patients with pre-bariatric disorder tend to develop BED in the postoperative period, which may be related to less weight loss. Subgroup tended to present vomiting due to weight change. The presence of these post-surgery disorders should be investigated, to identify who needs treatment.
Mack I (2016), Germany [13]	“To investigate the medium-term effects of LSG on mental health and eating behavior and their influence on weight loss by using a comprehensive interview-based assessment”.	After surgery, in the long term, depression, stress and eating disorders improve, BED being rare. Some patients experience disorders, along with depressive symptoms, greater stress and BMI, and less weight loss. Psychosocial improvement relates to weight loss, not surgery.
Hsu LKG (1996), USA [19]	“Examine what effect eating disturbances have on weight loss outcome after VBG”	AGB is not effective to change eating behavior or improve the patient’s psychiatric condition.
Latner JD (2004), New Zealand [17]	“To examine the prevalence of eating disturbances and psychiatric disorders among extremely obese patients before and after gastric bypass surgery and to examine the relationship between these disturbances and weight outcomes”	Presence of preoperative psychiatric disorders do not influence the outcome of bariatric surgery. More research is needed
Colles SJ (2008), Australia [18]	“This study prospectively assessed characteristics of BED, uncontrolled eating, NES and grazing, before, and 1 year after LAGB. We aimed to explore the nature and extent of change in these eating patterns following surgery”	More research is needed to optimize AGB results and improve postoperative psychological well-being
Scholtz S (2007), UK [16]	“To determine whether psychiatric profile was associated with long-term outcome”	The presence of psychiatric comorbidities should not be an impediment to performing bariatric surgery. The use of questionnaires should be considered mainly in the follow-up of patients with a psychiatric history
Smith KE (2019), USA [25]	“To [1] characterize LOCE and binge eating disorder (BED) over a 7-year period following bariatric surgery; [2] examine concurrent, prospective, and cumulative relationships between LOCE and weight loss; [3] assess whether these associations are moderated by surgery type; and [4] evaluate predictors of LOCE.”	LOCE and binge-eating can interfere with postoperative weight loss from bariatric surgery and must be constantly monitored
Powers PS (1999), USA [22]	“(1) to determine the prevalence of eating pathology in patients before bariatric surgery and at follow-up; (2) to assess the relationship of presurgical eating pathology to various measures of psychopathology; and (3) to assess the relationship between presurgical eating pathology and outcome”	There are no signs of a relation between preoperative disorders and postoperative vomiting episodes. During the first 6 months, all patients tend to lose more weight
Kalarchian MA (2019), USA [26]	“To report mental disorders through 7 years post surgery and examine their relationship with changes in weight and health-related quality of life”	Careful weight monitoring and post-operative mental disorders should optimize surgical results
Burgmer R (2005), Germany [24]	“The present study investigated the predictive value of three dimensions of eating behavior and disturbed eating on the course of weight after gastric restriction surgery”	Postoperative eating behavior influences surgery results more than preoperative behavior.
Rand CSW (1997), USA [14]	“To determine the prevalence of night-eating syndrome in the general population and among a new sample of obesity surgery patients”	Defined criteria, exacerbation factors and mitigation of their frequency and studies on the evolution of NES over time are needed
Luiz LB (2016), Brazil [20]	“To verify how the intensity of BE before the surgery and one year after the procedure, as well as the presence of BED, relate to the % EWL.”	The diagnosis of BED interferes negatively in weight loss
Kalarchian MA (2016), USA [21]	“To document changes in Axis I psychiatric disorders after bariatric surgery and examine their relationship with post surgery weight loss”	Preoperative disorders are not related to weight loss, unlike postoperative BED, which, although infrequent, is associated with less weight loss

LRYGB: Laparoscopic Roux-en-y gastric bypass. FA: food addiction. GBP: gastric by-pass. LAGB: laparoscopic adjustable gastric banding. RYGB: Roux-en-y gastric bypass. LBS: laparoscopic band surgery. VBG: vertical banded gastroplasty. BED: binge eating disorder. NES: night eating syndrome. LOCE: loss of control eating. LOC: loss of control. BE: binge eating. EWL: excess weight loss. AGB: adjustable gastric bandin.

**Table 3:** Experimental study main results.

Author, Publication Date and Country	Study Type	Evaluation Method	Type of Intervention	Eating Disorders before and after Surgery		Eating Symptoms before and after Surgery
Mack I (2016), Germany [13].	Non-randomized clinical trial	EDE TFEQ Other	SG	9 BED	1 BED	6 LOCE 39% Grazing Disinhibition and Feelings of Hunger reduced
Rand CSW (1997), USA [14].	Case control	Other	Other	30.6% of patients in the experimental group experienced NES.	Control Group: 1.5% NES Experimental Group: 27% NES	-

YFAS: Yale food addiction scale. EDE: eating disorder examination. TFEQ: three factor eating questionnaire. RYGB: Roux-en-y gastric bypass. AGB: adjustable gastric banding. SG: sleeve gastrectomy. FA: food addiction. BED: binge-eating disorder. NES: night-eating syndrome. LOCE: loss of control eating.

**Table 4:** Observational studies main results.

Author, Publication Date and Country	Type of Study	Evaluation Method	Type of Intervention	Eating Disorders before and after Surgery		Eating Symptoms before and after Surgery	
Larsen JK (2004), Netherlands [15].	Cross sectional	EDE BES Other	AGB	Pre-BED group: 55.9%	Short-term BED group: 31.9% Long-term BED group: 37.4%	Pre-group: 91 Emotional Eating; 93 External Eating; 92 Restrained Eating	Short-term group: 45 Emotional Eating; 48 External Eating; 48 Restrained Eating Long-term group: 102 Emotional Eating; 108 External Eating; 108 Restrained Eating
de Zwaan M (2009), Germany [23]	Cross sectional	EDE TFEQ Other	RYGB	15 BED (by EDE-BSV) 14 BED (by QEWP) 2 BN	-	45 Plugging (76.3%) 30 Dumping (50.8%) 15 SBE or LOCE (25.4%) 19 Picking/nibbling (32.2%) 37 Not weight-related vomiting (62.7%) 7 Weight-related vomiting (11.9%) 7 Nocturnal eating (11.9%)	15 LOCE 7 self-induced vomiting 37 vomiting for relief 7 symptoms of NES
Hsu LKG (1996), USA [19]	Cross sectional	EDE	AGB	19 Eating disorders (79.2%) 9 BED (37.5%) 5 BN (20.8%) 10 NES (42%), 8 of which are also BED/BN	5 BED (20.8%)5 BN (20.8%)8 previous BED or BN maintained the disease2 previous NES developed BED or BN	4 Self-induced vomiting	4 Self-induced vomiting
Latner JD (2004), New Zealand [17]	Prospective cohort	EDE	RYGB	BED: 48% 1 BN NES: 55%	BED: 0% BN: 0% NES: 2%	Vomiting:7% OBE: 20%	Vomiting: 5% OBE: 0%
Colles SJ (2008), Australia [18]	Prospective cohort	TFEQ SF-36 Other	AGB	18 BED (14%) 22 NES (17.1%)	4 BED (3.1%), with 2 being preoperative 10 NES (7.8%), with 4 being preoperative	LOCE: 31%	LOCE and Grazing: 20.2% Only Grazing: 5.9% Grazing had prevalence increased in 31%
Scholtz S (2007), UK [16]	Prospective cohort	EDE Other	AGB	12 BED (41%) 3 BN (10%) 1 AN (3%)	5 BED (17%) 0 BN (0%)	11 OBE (37%) 4 Symptoms of BED (13%)	4 Symptoms BED (13%)
Smith KE (2019), USA [25]	Prospective cohort	Other	RYGB AGB	BED: Total: 12.7% of 2157 RYGB: 12.1% of 1641 AGB: 14.6% of 516	BED (1 year): Total: 2.1% of 1774 RYGB: 1.3% of 1343 AGB: 4.5% of 431 BED (7 years): Total: 4% of 1049 RYGB: 3.3% of 812 AGB: 6.6% of 237	LOCE: Total: 35% of 2157 RYGB: 33.5% of 1641 AGB: 39.7% of 516	LOCE (1 year): Total: 24.6% of 1774 RYGB: 21.9% of 1343 AGB: 32.9% of 431 LOCE (7 years): Total: 26.4% of 1049 RYGB: 25.6% of 812 AGB: 29.1% of 237
Powers PS (1999), USA [22]	Prospective cohort	Other	Other	19 BED (16%) 12 NES (10%)	-	60 Symptoms BED (52%) 64 presented criteria for BED or NES (55%)	46 Occasional vomiting (79%) 19 weekly vomiting (33%)
Kalarchian MA (2019), USA [26]	Prospective cohort	SF-36 Other	RYGB AGB	RYGB: 8 BED (7.7%) 2 BN (1.9%) AGB: 2 BED (3%)	0 BED (0%) 0 BN (0%)	-	-
Burgmer R (2005), Germany [24]	Prospective cohort	TFEQ Other	AGB	BED: 7.4% BN: 3.4%	BED: 2%BN: 0.7%	Episodes of BED: 37.6% Grazing: 24.2%	Episodes of BED: 20.1% Grazing: 19.5%
Luiz LB (2016), Brazil [20]	Cross sectional	BES	RYGB	BED: 29.54%	BED: 7.58%	-	Elevation of BED symptoms: 13.63% Maintenance of BED symptoms: 6.83% Decrease in BED symptoms: 79.54%
Kalarchian MA (2016), USA [21]	Prospective cohort	Other	RYGB AGB	BED: 6.1% BN: 1.2%	BED: 3.1% BN: 0%	-	-

EDE: eating disorder examination. TFEQ: Three-Factor Eating Questionnaire. EDI: eating disorder inventory. BES: Binge-eating scale. SF-36: Medical Outcomes Study Short Form-36 Health Survey. YFAS: Yale food addiction scale. EDE-BSV: eating disorder examination-bariatric surgery version. QEWP: Questionnaire on Eating and Weight Patterns. RYGB: Roux-en-y gastric bypass. AGB: adjustable gastric banding. SG: Sleeve Gastrectomy. FA: food addiction. BED: binge-eating disorder. NES: night-eating syndrome. BN: bulimia nervosa. AN: anorexia nervosa. LOCE: loss of control eating.

**Table 5:** Reported study limitations.

Author, Publication Date and Country	Reported Study Limitations
Larsen JK (2004), Netherlands [15].	1. Population restricted to patients undergoing LAGB, does not allow generalization of procedures 2. Cross-sectional model, with comparison between groups (limits generalization and causal relation) 3. BES questionnaire used does not access the objective consumption of quantity of food in a short period of time
de Zwaan M (2009), Germany [23]	1. Small, non-consecutive sample (because there is a lot of refusal in the preoperative period to repeat the interview in the postoperative period) 2. Those who agreed to be interviewed in the postoperative period may not represent the population as a whole 3. Interview based on EDE-BSV was not conducted in the pre and postoperative period 4. Relatively short follow-up
Mack I (2016), Germany [13]	1. Proportionally significant loss of follow-up (considering obese group) 2. Final sample of 66% of the initial sample 3. Depression accessed only by validated questionnaires, without a structured interview, limiting the validity of the results
Hsu LKG (1996), USA [19]	1. Retrospective cross-sectional design 2. Short study duration 3. Small sample size
Latner JD (2004), New Zealand [17]	1. Retrospective assessment of eating disorders 2. Absence of men in the sample 3. Use of self-reported methods, with follow-up interviews via telephone and face-to-face measurements 4. Short study duration
Colles SJ (2008), Australia [18]	1. Use of self-report survey and telephone interview for assessment of postoperative eating behavior 2. Overlapping of groups and absence of agreed group definitions
Scholtz S (2007), UK [16]	1. Small sample size 2. Significant number of cases excluded from the analysis due to the absence of psychiatric evaluation 3. Retrospective assessment of eating disorders
Smith KE (2019), USA [25]	1. Evaluation methodology of LOCE and binge eating can interfere with the results obtained 2. Use of self-reported questionnaires 3. The proportion of AGB cases in the sample may not correspond to national averages
Powers PS (1999), USA [22]	No limitations reported by the authors
Kalarchian MA (2019), USA [26]	1. Possible risk of attrition or self-selection bias 2. Limited statistical power for some analyses due to sample size and loss of follow-up 3. According to the methodology used, the last month of evaluation may not represent the total number of diagnoses from that period. 4. There was no justification for the prescriptions used by patients during the study
Burgmer R (2005), Germany [24]	1. Pre-selection of patients 2. Exclusive results for restrictive surgeries
Rand CSW (1997), USA [14]	Self-selection of patients in the case group and therefore the prevalence of NES was higher
Luiz LB (2016), Brazil [20]	1. The study involved only one center 2. Involvement mainly of Caucasian women, making it difficult to extrapolate the data to the general population. 3. The diagnosis of BED by BES tends to be very sensitive and unspecific, overestimating it. 4. Possibly insufficient follow-up to clearly evaluate how BED variation interferes with weight loss
Kalarchian MA (2016), USA [21]	1. Possible selection bias due to self-selection to participate in the study or due to dropout 2. Limited statistical power for some analyses 3. Underestimation of disorders by applying DSM-IV criteria, which do not detect subclinical disorders 4. The evaluation period was only in 2 and 3 years after surgery, therefore, if patients develop the disorder in other periods, it will not be detected. 5. Results of RYGB and LAGB only

YFAS: Yale food addiction scale. EDE-BSV: eating disorder examination-bariatric surgery version. DSM-IV: Diagnostic and Statistical Manual of Mental Disorders-IV. BED: binge-eating disorder. FA: Food addiction. NES: night-eating syndrome. LOCE: loss of control eating. RYGB: Roux-en-y gastric bypass. AGB: adjustable gastric banding.

## Data Availability

The authors confirm that the data supporting the findings of this study are available within the article.
